# Case report: Acute calcific discitis with intravertebral disc herniation in the dorsolumbar spine

**DOI:** 10.4103/0971-3026.69360

**Published:** 2010-08

**Authors:** Puneet Mittal, Kavita Saggar, Parambir Sandhu, Kamini Gupta

**Affiliations:** Department of Radiodiagnosis, Dayanand Medical College & Hospital, Ludhiana, Punjab, India

**Keywords:** Acute, calcific, discitis, dorsolumbar, MR

## Abstract

Acute calcific discitis is a rare but well-known condition of unknown etiology. In symptomatic cases, the most common site is the cervical spine. We describe the CT scan and MRI findings in a symptomatic patient, with a lesion in the dorsolumbar spine.

## Introduction

Acute calcifc discitis is a rare condition. When symptomatic, it can be mistaken for infection.[1] Most of the symptomatic cases present in the cervical spine.[1–3] We present the CT scan and MRI findings in a patient who had involvement of the dorsolumbar spine, with associated intravertebral disc herniation.

## Case Report

**Figure 1:**
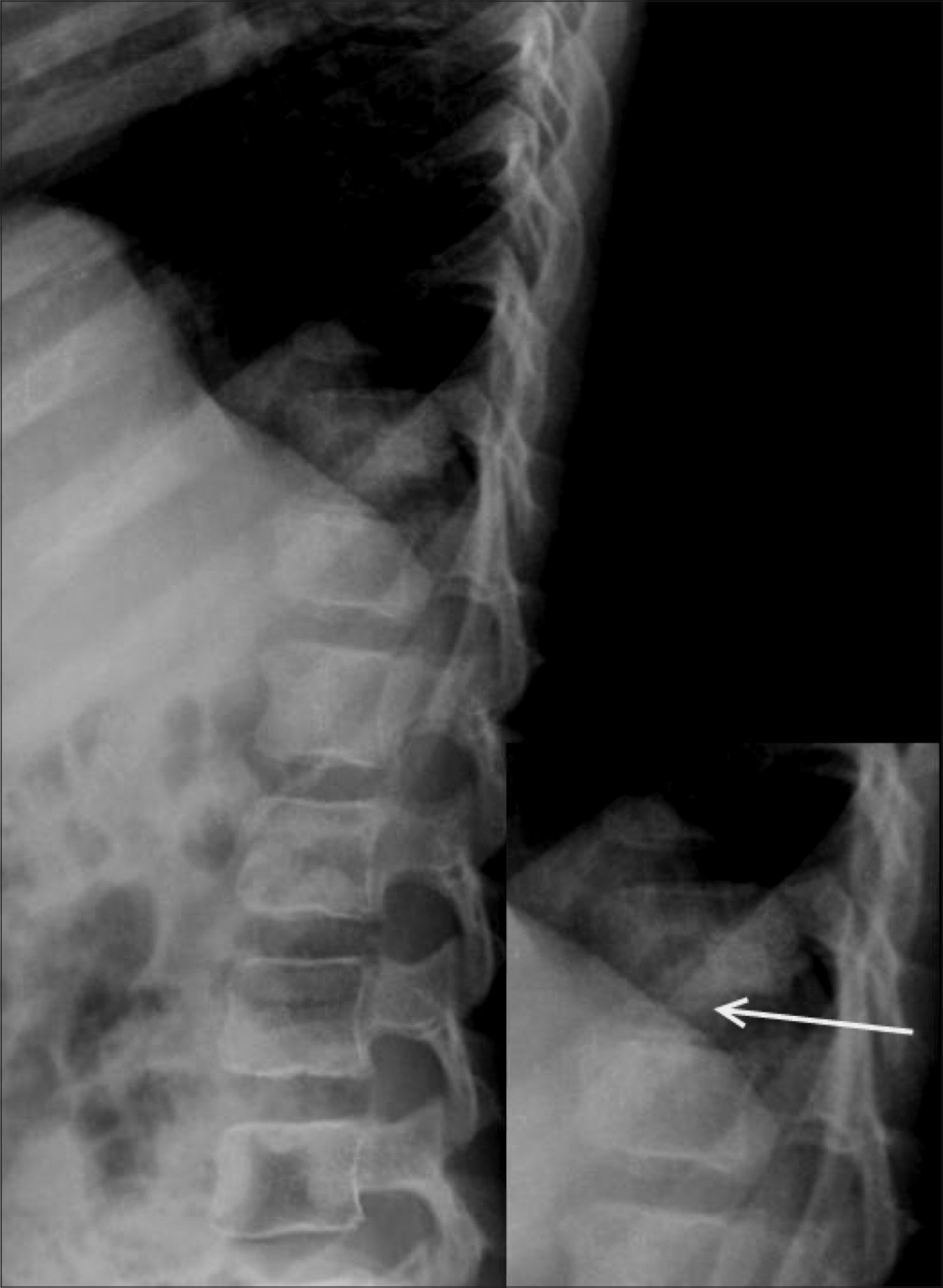
Lateral dorsolumbar spine radiograph obtained a day after the MRI scan shows calcification of the nucleus pulposus (arrow)

A 10-year-old boy presented with a 2-week history of pain in the lower back following a yoga session in school. The pain had gradually worsened over the last 5 days. The patient was afebrile. The total white blood cell (WBC) count was normal. The erythrocyte sedimentation rate (ESR) was raised (52 mm/h). The Mantoux test was negative. A radiograph obtained elsewhere and repeated a day after the MRI [Figure 1], showed calcification of the D12-L1 intervertebral disc.

**Figure 2 (A-D):**
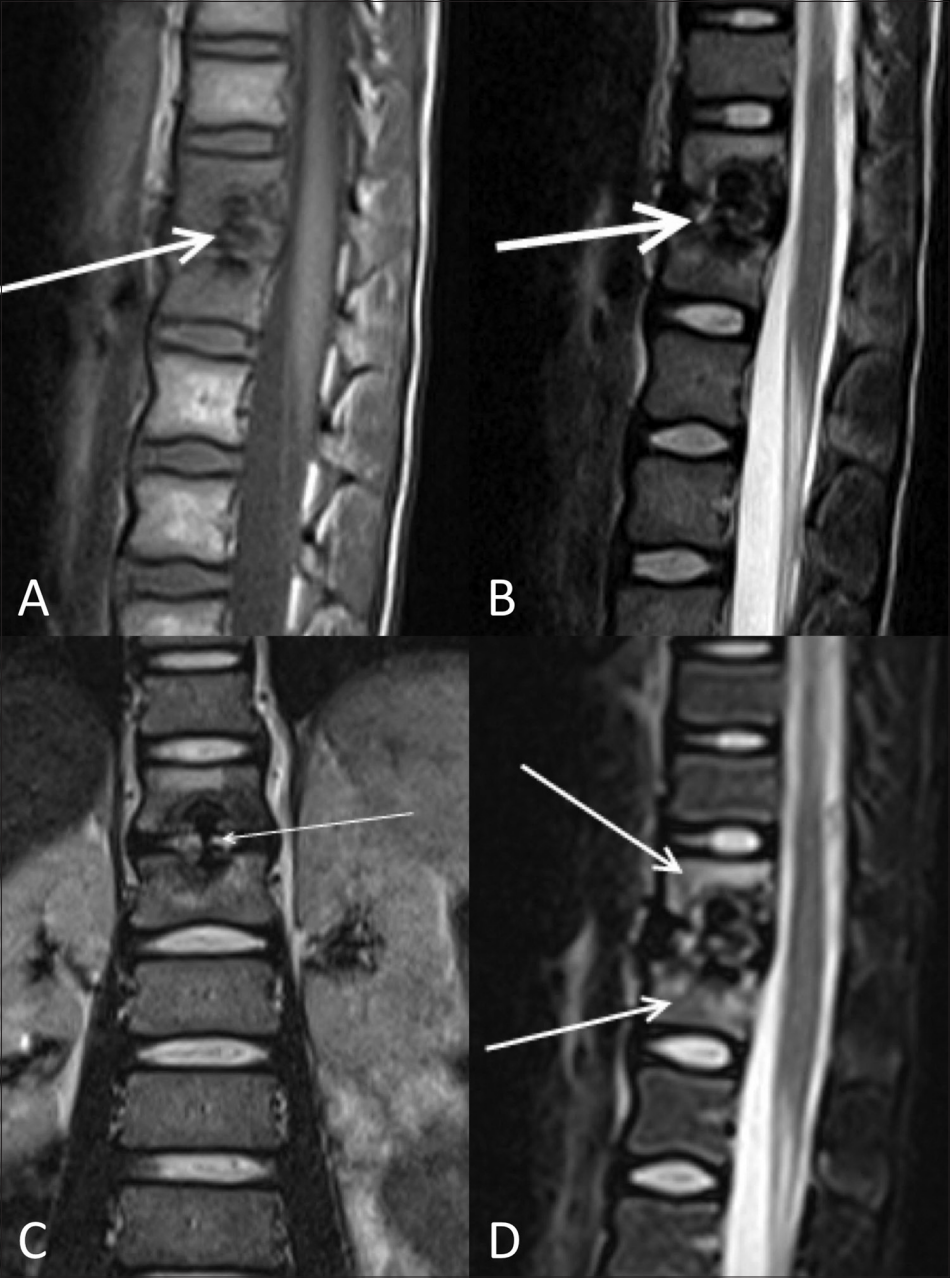
Sagittal T1W (A), sagittal T2W (B), and coronal T2W (C) MRI images show swelling of the nucleus pulposus with hypointense signal (arrows) on both the T1W and T2W images, due to calcification. The coronal T2W image (C) clearly shows the dumbbell-shaped calcification with intravertebral herniation without end plate destruction (arrow). Sagittal STIR MRI image (D) shows evidence of marrow edema (arrows) in the bodies of the adjacent vertebrae

**Figure 3:**
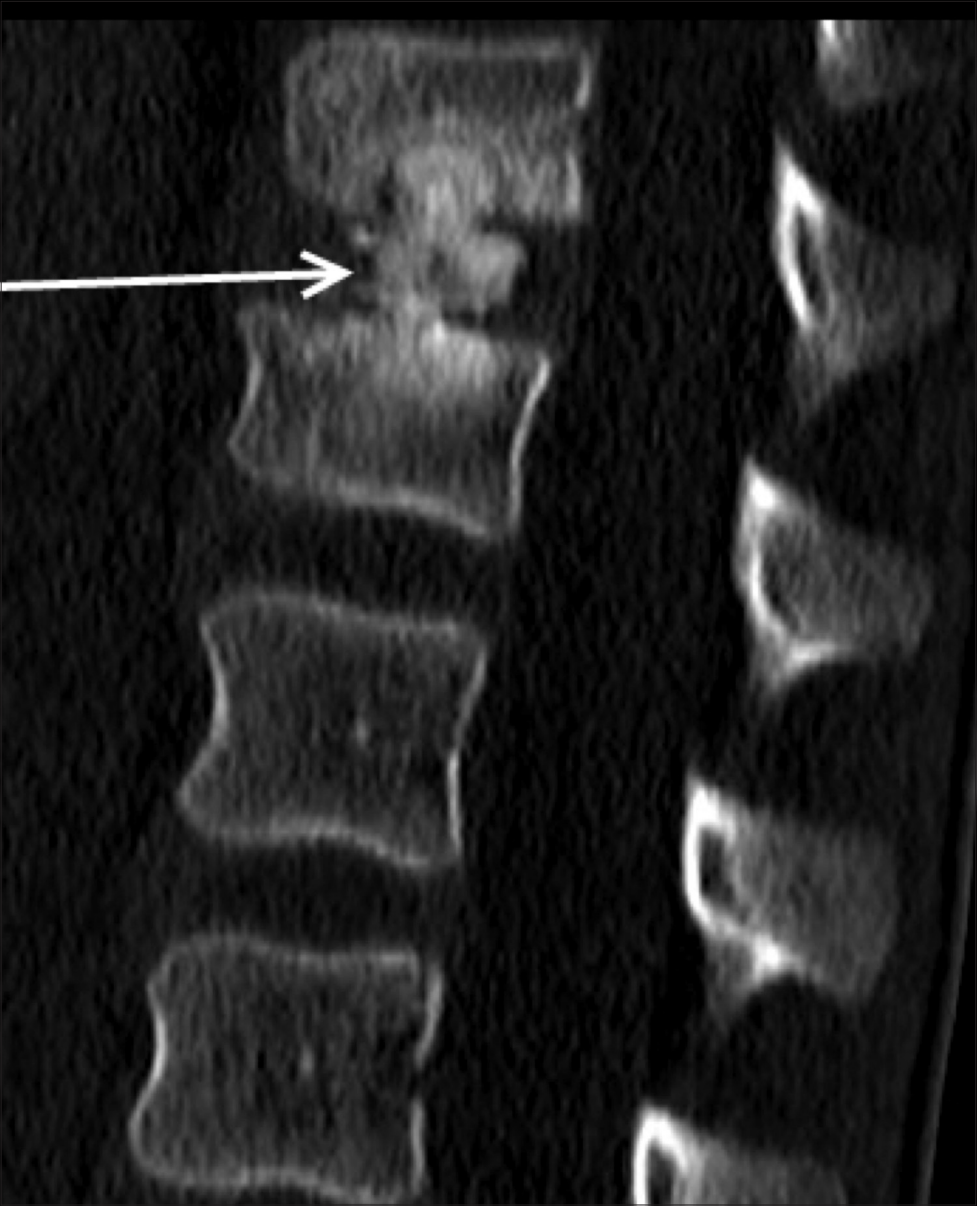
Reformatted sagittal CT scan shows swelling and calcification of the D12/L1 nucleus pulposus with intravertebral herniation (arrow), without endplate destruction

**Figure 4:**
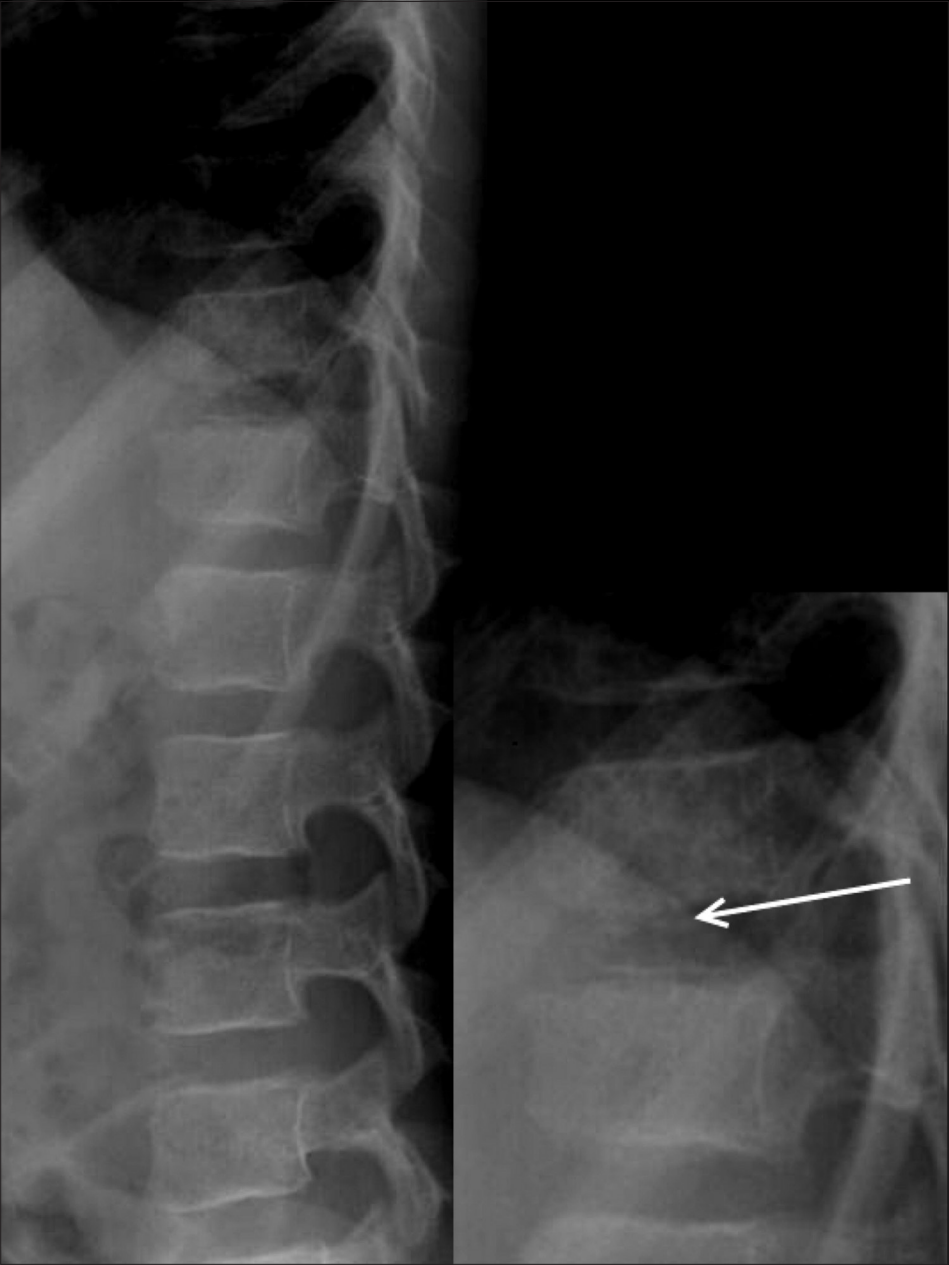
Lateral dorsolumbar spine radiograph obtained 2 weeks after presentation shows partial resolution of the calcification (arrow)

MRI showed hypointense signal in the D12-L1 intervertebral disc on T1W [Figure 2A] and T2W [Figure 2B and C] images. There was evidence of marrow edema in the bodies of the D12 and L1 vertebrae on the STIR images [Figure 2D]. CT scan revealed calcification of the nucleus pulposus, with intravertebral herniation at the D12-L1 level, causing a smooth indentation of the endplates without any evidence of erosions [Figure 3]. The patient was treated conservatively. He improved significantly over 2 weeks. A follow-up radiograph obtained after 2 weeks showed partial resolution of the calcification [Figure 4].

## Discussion

Acute calcific discitis is a rare condition. Till 1988, there had been less than 130 reported cases of this condition.[1] Many more cases have been reported since then, but the actual incidence of this condition is unknown since many cases are asymptomatic and go unnoticed.[3] Most patients present between 5–10 years of age. There is a male preponderance.[1] Most symptomatic cases have involvement of the cervical spine (about 70% of symptomatic cases) and present with pain and torticollis. Most of those who have thoracic spine involvement are asymptomatic.[1,2] Sometimes, fever, raised WBC count, and increased ESR may be present, which may falsely suggest an infective etiology.[4–6] Our patient was afebrile and his WBC count was normal, though the ESR was raised. The natural history is of spontaneous resolution of symptoms usually within weeks, though this may sometimes take upto 6 months.[2]

The cause of the intervertebral disc calcification remains unknown. Trauma has been implicated as a possible cause; however, a history of trauma is not always present.[6] Recently, interruption of blood supply – which could be secondary to a variety of insults like trauma, inflammation, or vasculitis – has been suggested as a possible etiology.[7] However, there is no firm evidence to support this. Pediatric intervertebral disc calcification differs from that in adults in many respects. In adults, calcification involves the annulus fibrosus, is permanent, and most commonly is seen in the thoracolumbar region. On the other hand, in the pediatric population, it involves the nucleus pulposus, is transient, and most commonly involves the cervical region.[5] The calcification diminishes or completely resolves on follow up in the majority of the patients, but sometimes may persist for months to years, even after the symptoms have resolved.[5,7]

The initial event is swelling of the disc, which is best appreciated on MRI; this may or may not be associated with visible calcification on radiographs. Calcification presents with low signal in the intervertebral disc on both T1W and T2W images. There may be associated marrow edema in the adjacent vertebral bodies,[7,8] which can be mistaken for infectious spondylodiscitis. However, in infection, the disc space is reduced rather than increased. Moreover, endplate destruction, which occurs in infection, is not seen in acute calcific discitis. CT scan is useful for demonstrating intact endplates as well as the calcification if there is any doubt on MRI.

In conclusion, acute calcific discitis is an uncommon cause of back pain in children. It usually involves the cervical spine, where it presents with pain and torticollis. However, thoracolumbar calcifications may also be symptomatic, as in our case. The presence of marrow edema in adjacent vertebrae should not be mistaken for infection. The presence of disc swelling and calcification along with an intact endplate should suggest this diagnosis and prevent unnecessary diagnostic workup.
